# Seroprevalence of Pandemic (H1N1) 2009 in Pregnant Women in China: An Observational Study

**DOI:** 10.1371/journal.pone.0017995

**Published:** 2011-03-18

**Authors:** Xiang Huo, Xian Qi, Fenyang Tang, Rongqiang Zu, Liang Li, Bin Wu, Yuanfang Qin, Hong Ji, Jianguang Fu, Shenjiao Wang, Hua Tian, Zhibin Hu, Haitao Yang, Minghao Zhou, Hua Wang, Fengcai Zhu

**Affiliations:** 1 Jiangsu Provincial Center for Disease Prevention and Control, Nanjing, China; 2 College of Public Health, Nanjing Medical University, Nanjing, China; National University of Singapore, Singapore

## Abstract

**Background:**

We investigated the seropositive rates and persistence of antibody against pandemic (H1N1) 2009 virus (pH1N1) in pregnant women and voluntary blood donors after the second wave of the pandemic in Nanjing, China.

**Methodology/Principal Findings:**

Serum samples of unvaccinated pregnant women (n = 720) and voluntary blood donors (n = 320) were collected after the second wave of 2009 pandemic in Nanjing. All samples were tested against pH1N1 strain (A/California/7/2009) with hemagglutination inhibition assay. A significant decline in seropositive rates, from above 50% to about 20%, was observed in pregnant women and voluntary blood donors fifteen weeks after the second wave of the pandemic. A quarter of the samples were tested against a seasonal H1N1 strain (A/Brisbane/59/2007). The antibody titers against pH1N1 strain were found to correlate positively with those against seasonal H1N1 strain. The correlation was modest but statistically significant.

**Conclusions and Significance:**

The high seropositive rates in both pregnant women and voluntary blood donors suggested that the pH1N1 virus had widely spread in these two populations. Immunity derived from natural infection seemed not to be persistent well.

## Introduction

In April 2009, an emergent pandemic influenza strain, now called pandemic (H1N1) 2009 (pH1N1), infected thousands of persons in Mexico and the United States and then spread rapidly throughout the globe [1]–[4]. The first case in Mainland China was identified on 11 May 2009, while in Nanjing (the capital city of Jiangsu province with 6.44 million people) it was in mid-June 2009. The epidemic in Nanjing reached its peak in mid-September and late November, and attenuated to baseline levels by late December, 2009. As in previous influenza epidemics and pandemics, pregnant women appeared to be at higher risk of serious consequences following infection with pH1N1virus [5]–[11]. In fact, pregnant women accounted for 25% of serious cases of pH1N1reported to the Jiangsu Provincial Center for Disease Prevention and Control (JSCDC). Therefore, it is critical to investigate the seropositive rates and antibody persistence in pregnant population which may provide valuable information for decision-making on immunization strategies and other prevention measures for pregnant women. Several cross sectional studies had revealed the seroprevalence of antibody against pandemic (H1N1) 2009 strain in the general population after the pandemic [12]–[14]. However, data on seroprevalence of pH1N1 antibody in pregnant women were limited, especially the antibody persistence after the pandemic. The study aimed to describe the seropositive rates and persistence of antibody against pH1N1in pregnant women after the second wave of the pandemic.

## Methods

### Ethics statement

Serum samples were collected from the residual sera used for routine check-up of pregnant women, and from stored sera of voluntary blood donors without additional collection. All the samples were collected and analyzed anonymously. Written informed consents were received from all participants. The study was approved by the Ethics Committee of JSCDC.

### Samples collection

**Figure 1:**
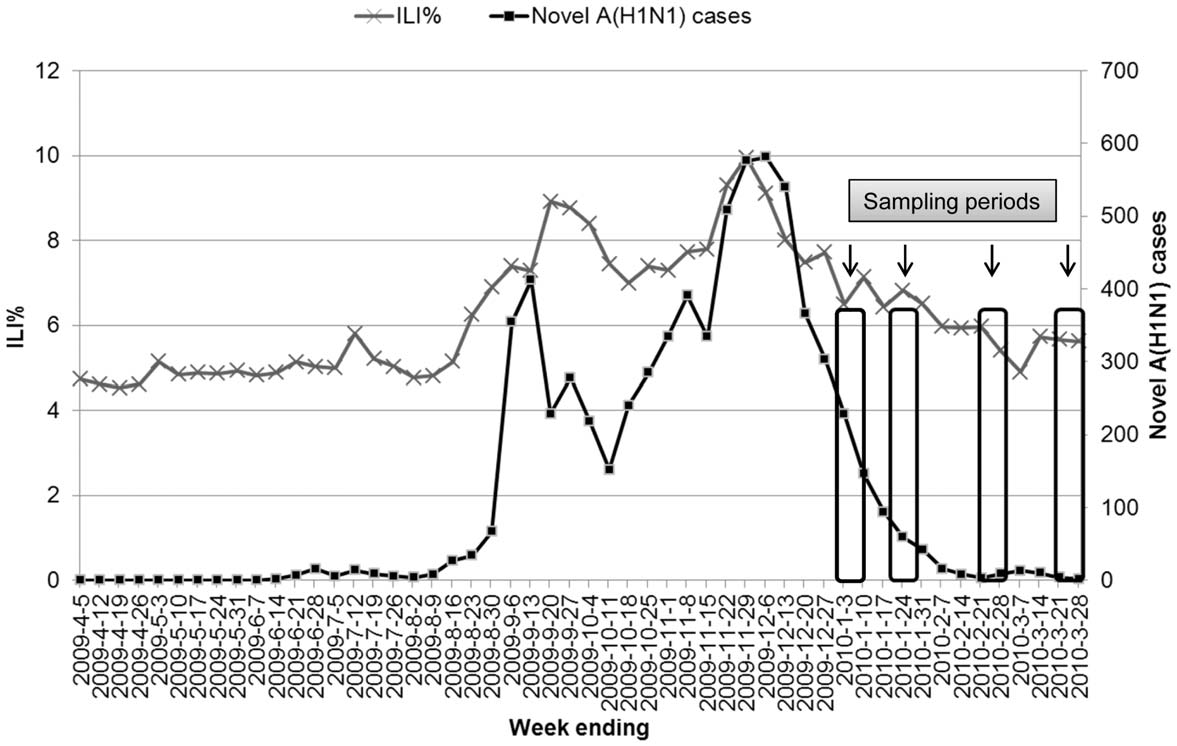
Sampling times and epidemic curve. Sampling times of serologic survey (Jan 1–Mar 28, 2010) shown relative to epidemic curve of pH1N1 cases and percent of ILI (influenza-like illness, ILI %) accounted for out-patient and emergency cases.

The samples were collected four times after the second wave of pandemic (H1N1) 2009, i.e. on January 1–10, January 20–28, February 20–28 and March 20–28, 2010 (Figure 1). A total of 1040 serum samples were collected, of which 720 samples (180 each time) from pregnant women were collected anonymously in two large maternity and children hospitals in Nanjing. And 320 samples (80 each time) from voluntary blood donors were randomly collected anonymously as control in Jiangsu Province Blood Center in Nanjing. Demographic characteristics such as age, gender and gestational week, were collected. The ages of enrolled pregnant women were equally distributed among the four sampling times. The gestational weeks were taken into account to ensure that the participants were pregnant at the time of virus circulation. All participants did not receive pH1N1 and seasonal influenza vaccines before bleeding.

### Hemagglutination inhibition (HAI) assay

All serum samples were tested with HAI assay against pH1N1 strain (A/California/7/2009) and a quarter of samples was tested against a seasonal H1N1 strain (A/Brisbane/59/2007), which was conducted as previously described [15], [16]. To inactivate non-specific inhibitors, all serum samples were first treated with receptor destroying enzyme (Cholera filtrate, GIGMA) and then serially diluted into 2-fold dilution starting with a 1∶10 dilution in V-bottom 96-well micro titer plates. An equal volume of virus dilution containing about 4HA units/50 µl was added to each well. The plates were incubated at room temperature for 50 min followed by addition of freshly prepared 1% chicken erythrocyte, and then incubated at 25°C for 30 min. The HAI titer was determined by the reciprocal of the last dilution showing no agglutination of erythrocyte. Both positive and negative controls were included in each plate. Samples with HAI titer ≥1∶40 were considered seropositive. It was generally accepted that serum HAI titers of 40 are associated with at least a 50% reduction in risk of infection or disease with seasonal influenza viruses in human population [12].

### Sample Size

Sample size calculations were performed by EpiCalc (v1.02) based on an estimated seroprevalence of 30%, which indicated that 80 samples would be required per time to detect seroprevalence +/−10% within a 95% confidence interval. The precision would increase to +/−6.7% if the sample size were more than 180.

### Statistical analysis

Kolmogorov-Smirnov test was used to test the normality of data. The differences of non-normal distribution data were compared by Kruskal-Wallis test. Spearman correlation coefficients were calculated to describe the correlation between antibody titers against pH1N1 and seasonal H1N1 viruses. Cochran-Armitage test was used for trend test across the four sampling times. Chi square tests were used to compare the differences of seropositive rates between pregnant women and voluntary blood donors among the four sampling times. All the statistical analysis was performed with Statistical Analysis System software (9.1.3; SAS Institute, Cary, NC, USA).

## Results

A total of 720 pregnant women and 320 voluntary blood donors were included in final analysis. There were no statistically significant differences in age among the four sampling times (***P*** = 0.808). The gestational weeks in the women participants showed an increase trend across the sampling times (Table 1). In fact, 98.06% of the enrolled pregnant women had been pregnant prior to the pandemic and the remained became pregnant during the first 1–2 weeks of the pandemic. In voluntary blood donors, the median and interquartile range of ages were 24 years and 21–30 years, respectively, and the sex ratio was 1.02.

**Table 1:** Ages and gestational weeks of pregnant women when serum samples were collected.

Sampling times	Weeks after the second wave	Age (median, quartiles), years	Gestational weeks (median, quartiles)
Jan 1–10	4 weeks	28 (26–30)	35 (31–38)
Jan 20–28	7 weeks	28 (27–31)	36 (32–38)
Feb 20–28	11 weeks	28 (27–30.75)	37 (32–40)
March 20–28	15 weeks	28 (26–31.75)	37 (31.25–40)
*P* *		0.808	0.004

*Kruskal-Wallis Test.

**Figure 2:**
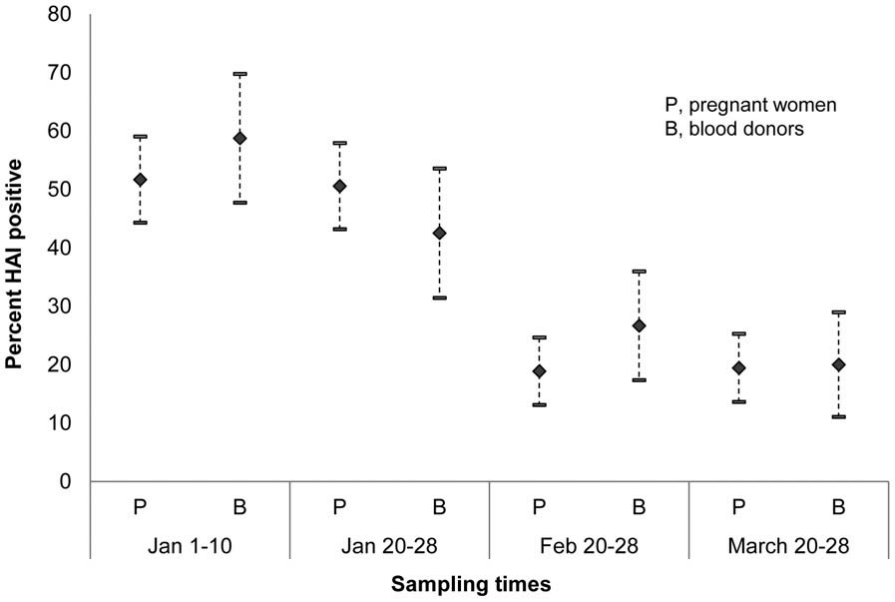
Seroprevelance in pregnant women and voluntary blood donors against pH1N1. Seropositive rates (HAI≥1∶40) of antibodies against pH1N1 in pregnant women and voluntary blood donors at the 4 sampling times after the second wave of pandemic in Nanjing, China.

At the first and second sampling times in January, about 4 and 7 weeks after the second wave, the seropositive rates against pH1N1 were 51.67% (95%CI, 44.30%–59.04%) and 50.56% (95%CI, 43.18%–57.93%) in pregnant women, respectively, while 58.75% (95%CI, 47.73%–69.77%) and 42.50% (95%CI, 31.43%–53.57%) in voluntary blood donors, respectively. The seropositive rates showed no significant differences between the two populations. The seropositive rates declined significantly (***P*** for trend <0.0001) afterward. At the third and fourth sampling times in February and March, about 11 and 15 weeks after the second wave, the seropositive rates were 18.89% (95%CI, 13.12%–24.66%) and 19.44% (95%CI, 13.61%–25.28%) in pregnant women, respectively, while 26.67% (95CI, 17.35%–35.98%) and 20.00% (95%CI, 11.04%–28.96%) in voluntary blood donors, respectively. No significant differences were found either between pregnant women and voluntary blood donors (Table 2, Figure 2).

**Table 2:** Seropositive rates of antibodies against pH1N1 in pregnant women and voluntary blood donors at the 4 sampling times.

Sampling times	Pregnant (%, 95%CI)	Voluntary blood donors (%, 95%CI)	*P * *
Jan 1–10	51.67 (44.30–59.04)	58.75 (47.73–69.77)	0.29
Jan 20–28	50.56 (43.18–57.93)	42.50 (31.43–53.57)	0.23
Feb 20–28	18.89 (13.12–24.66)	26.67 (17.35–35.98)	0.14
March 20–28	19.44 (13.61–25.28)	20.00 (11.04–28.96)	0.92
*P* trend†	<0.0001	<0.0001	

Seropositive rates, Proportion of titers of 1∶40 or more (%).

*Chi square tests.

† Cochran-Armitage test.

To detect possible cross-reaction between pH1N1and seasonal H1N1 viruses, serum samples collected during Jan 1–10 were tested simultaneously against a seasonal H1N1 influenza strain, A/Brisbane/59/2007, which was recommended by the World Health Organization for vaccine manufacturers in the last 3 influenza seasons [17]–[19]. The HAI titers against pH1N1 strain were found to correlate positively with those against seasonal H1N1 strain. The correlation was modest but statistically significant (R = 0.36, ***P***<0.0001 for Spearman test).

## Discussion

The emergence of pH1N1 strain presented a public health challenge. The investigation on epidemiological features was critical for influenza prevention and control. Serologic survey was a useful tool to understand the seropositive rates and antibody persistence after natural infection, especially in some populations at high risk, such as pregnant women. In this study, the seropositive rates and persistence of antibodies in unvaccinated pregnant women and voluntary blood donors were determined in the post-pandemic period in Nanjing, China, contributing to our knowledge on immune status in the pregnant women and voluntary blood donors.

Four weeks after the second wave of the pandemic in Nanjing, the seropositive rates remained somewhat high in pregnant women and voluntary blood donors, which were similar to those in a previous report in Norway (44.9% overall, 65.3%highest) [20]. However, the seropositive rates in our study were much higher than those in pregnant women (15.7% overall, 20.8%highest, n = 296) in Manitoba of Canada after the first wave of pandemic (August 2009) [21], in general population (14.5%) in United Kingdom after the first wave of pandemic [13], and in Pittsburgh-area residents (21%) in USA one month after the second wave (mid-November and early December 2009) [12]. One of the possible reasons for the difference might be the different infection rates between the first and second wave of pandemic. The age composition of study population could also impact the seropositive rates since young adults were at higher risk of infection with pH1N1 virus. In addition, the susceptibility to the pandemic virus may relate to ethnic, health behavior and vaccination history. And the pre-pandemic baseline level of antibody and the sensitivity of HAI assay could be the possible reasons [22].

Deng et al conducted a serological survey in 710 residents in Beijing and found a seropositive rate of 14.0% overall but a lower rate of 4.5% in individuals aged 60 years or more by comparison with individuals in other age groups (0–5, 6–17, 18–59 years) [23], which were significantly different from our findings and other seroepidemiological studies [12], [13], [24]. At least two reasons could account for this diversity. Firstly, most of the participants in the study of Deng et al were recruited from outpatients with various clinical conditions, where a selection bias might produce. Secondly, the second wave of pandemic in China was from November 22 to November 29, 2009 according to China influenza monitoring weekly written by China CDC (http://www.cnic.org.cn/chn/). And in Nanjing, the second wave was from November 30 to December 6, 2009 (Figure 1). The sampling time of Deng et al was from 29 November to 5 December, 2009, by which the duration of antibody response against natural infection might not be long enough.

At present, data on persistence of antibodies against pH1N1virus due to natural infection were limited. The results derived from our study may address the gap. We found that the seropositive rates derived from natural infection declined significantly in both pregnant women and voluntary blood donors after the second wave of the pandemic, from above 50% to about 20% in an 11-week interval. Prior to the pandemic, unvaccinated population had very low baseline HAI titers against the emergent strain pandemic (H1N1) 2009. Song et al found that pre-vaccination low HAI titer (<1∶40) was associated with early decline of HAI titer [25]. In a previous survey conducted in regions surrounding Nanjing in November 2008 by JSCDC, the seropositive rate (HAI titers ≥1∶40) of antibody against pandemic H1N1 was found to be only 3.64% [26]. The low baseline HAI titers in naïve population might account for the early decline of pH1N1 titers in this study. Similarly, the HAI titers against H5N1 strain were very low in naive population as well. It was found that individuals' antibody levels also significantly declined several months after the second dose of H5N1 vaccine [27]. However, a third dose of the same vaccine could significantly boost immune responses [27]. Accordingly, vaccination with the same pH1N1strain would be needed for the naturally infected population in our study to acquire enough immunity. In addition, memory T cells also play a role in the cellular immune response to secondary infection [28]: flu-specific CD8+T-lymphocyte responses are important in decreasing disease severity and facilitating viral clearance [29], and CD4+ T cells are able to mediate potent antibody-independent antiviral immunity [28], [30]. Thus, the impact of the significant decline in seropositive rates of antibodies on the protection against secondary infection of the naturally infected population needs further investigation.

The protein sequence similarity of the pandemic H1N1 2009 and human seasonal influenza H1N1 is about 80% [31]. In our study, a modest but statistically significant correlation between HAI titers of these two strains was found, indicating a possible cross-reaction. In a previous survey conducted in regions surrounding Nanjing prior to the pandemic, the overall seropositive rates of antibodies against pH1N1 were 3.64%, and 0, 6.20%, 3.75% and 5.06% in subgroups of 3–11years, 12–17years, 18–60years and ≥60 years, respectively [26]. These pre-pandemic data also supported possible cross-reaction. In a previous study conducted in a population in U.S., authors also found a modest correlation between seasonal A/Brisbane/59/2007 H1N1 and pH1N1 [12]. In addition, Chen et al. found antibodies derived from natural infection with pH1N1 showed cross reaction with seasonal influenza viruses. There was a four-fold or greater increase in cross-reactive antibody titers to seasonal A/Brisbane/59/2007 H1N1 in 20% of RT-PCR confirmed pandemic (H1N1) 2009 infected participants [32].

In conclusion, the seropositive rates and persistence of antibodies in unvaccinated pregnant women and voluntary blood donors were determined after the second wave of 2009 pandemic in Nanjing, China, contributing to our understanding on the changes of immunity derived from natural infection. Our findings of high seropositive rates in pregnant women and voluntary blood donors, suggested that pandemic (H1N1) 2009 strain had widely spread in these populations. Further efforts would be needed to investigate the impact of declined antibody level on secondary infection of the same strain.
